# Supplementary material for: Tunable magnetoplasmonics in lattices of Ni/SiO2/Au dimers
Source: Sci Rep. 2019 Jul 9;9:9907. doi: 10.1038/s41598-019-46058-2 (PMC6617622; doi:10.1038/s41598-019-46058-2)
Supplement: Supplementary file 1 — Supplementary Information [file 41598_2019_46058_MOESM1_ESM.pdf]

Tunable magnetoplasmonics in lattices of Ni/SiO$_2$/Au dimers

Sara Pourjamal[1], Mikko Kataja[1,2], Nicolò Maccaferri[3], Paolo Vavassori[4], Sebastiaan van Dijken[1,*]

[1] NanoSpin, Department of Applied Physics, Aalto University School of Science, P.O. Box 15100, FI-00076 Aalto, Finland
[2] Institut de Ciència de Materials de Barcelona (ICMAB-CSIC), Campus de la UAB, Bellaterra, Catalonia, Spain
[3] Physics and Materials Science Research Unit, Université du Luxembourg, L-1511 Luxembourg, Luxembourg
[4] CIC nanoGUNE, E-20018 Donostia-San Sebastian, Spain; and Ikerbasque, Basque Foundation for Science, E-48013 Bilbao, Spain
* sebastiaan.van.dijken@aalto.fi

1. Effective polarizability of a two dimensional lattice under normal incidence radiation

To derive the effective lattice polarizability, we start from Eq. 11 in the main manuscript. Matrix inversion and subtraction gives

$$\alpha_{\mathrm{eff}} = \begin{bmatrix} \frac{\alpha_{\mathrm{yy}}}{\alpha_{\mathrm{xx}}\alpha_{\mathrm{yy}}-\alpha_{\mathrm{xy}}^2} - S_{\mathrm{x}} & -\frac{\alpha_{\mathrm{xy}}}{\alpha_{\mathrm{xx}}\alpha_{\mathrm{yy}}-\alpha_{\mathrm{xy}}^2} \\ \frac{\alpha_{\mathrm{xy}}}{\alpha_{\mathrm{xx}}\alpha_{\mathrm{yy}}-\alpha_{\mathrm{xy}}^2} & \frac{\alpha_{\mathrm{xx}}}{\alpha_{\mathrm{xx}}\alpha_{\mathrm{yy}}-\alpha_{\mathrm{xy}}^2} - S_{\mathrm{y}} \end{bmatrix}^{-1} \quad \text{(S1)}$$

Using $\alpha_{\mathrm{xx,yy}} \gg \alpha_{\mathrm{xy}}$, we find

$$\alpha_{\mathrm{eff}} = \begin{bmatrix} \frac{1}{\alpha_{\mathrm{xx}}} - S_{\mathrm{x}} & -\frac{\alpha_{\mathrm{xy}}}{\alpha_{\mathrm{xx}}\alpha_{\mathrm{yy}}} \\ \frac{\alpha_{\mathrm{xy}}}{\alpha_{\mathrm{xx}}\alpha_{\mathrm{yy}}} & \frac{1}{\alpha_{\mathrm{yy}}} - S_{\mathrm{y}} \end{bmatrix}^{-1} \quad \text{(S2)}$$

Inversion of this matrix gives

$$\alpha_{\mathrm{eff}} = \frac{1}{(\frac{1}{\alpha_{\mathrm{xx}}}-S_{\mathrm{x}})(\frac{1}{\alpha_{\mathrm{yy}}}-S_{\mathrm{y}})} \begin{bmatrix} \frac{1}{\alpha_{\mathrm{yy}}} - S_{\mathrm{y}} & \frac{\alpha_{\mathrm{xy}}}{\alpha_{\mathrm{xx}}\alpha_{\mathrm{yy}}} \\ -\frac{\alpha_{\mathrm{xy}}}{\alpha_{\mathrm{xx}}\alpha_{\mathrm{yy}}} & \frac{1}{\alpha_{\mathrm{xx}}} - S_{\mathrm{x}} \end{bmatrix} =$$

$$\begin{bmatrix} \frac{1}{\alpha_{\mathrm{xx}}} - S_{\mathrm{x}} & \frac{\alpha_{\mathrm{xy}}}{\alpha_{\mathrm{xx}}\alpha_{\mathrm{yy}}\left(\frac{1}{\alpha_{\mathrm{yy}}}-S_{\mathrm{xx}}\right)\left(\frac{1}{\alpha_{\mathrm{xx}}}-S_{\mathrm{yy}}\right)} \\ -\frac{\alpha_{\mathrm{xy}}}{\alpha_{\mathrm{xx}}\alpha_{\mathrm{yy}}\left(\frac{1}{\alpha_{\mathrm{yy}}}-S_{\mathrm{xx}}\right)\left(\frac{1}{\alpha_{\mathrm{xx}}}-S_{\mathrm{yy}}\right)} & \frac{1}{\alpha_{\mathrm{yy}}} - S_{\mathrm{y}} \end{bmatrix} \quad \text{(S3)}$$

Equations 12 and 13 in the main manuscript correspond to the diagonal and off-diagonal terms of this matrix.

2. Near-field coupling between dipoles inside dimers

Coupling between dipoles in the Ni and Au disks of vertical dimers is described by the dyadic Green's function ***G*** (Eq. 7 in the main manuscript). Supplementary Fig. 1 shows the calculated real and imaginary parts of ***G*** and it's magnitude as a function wavelength for different SiO_2 layer thickness.

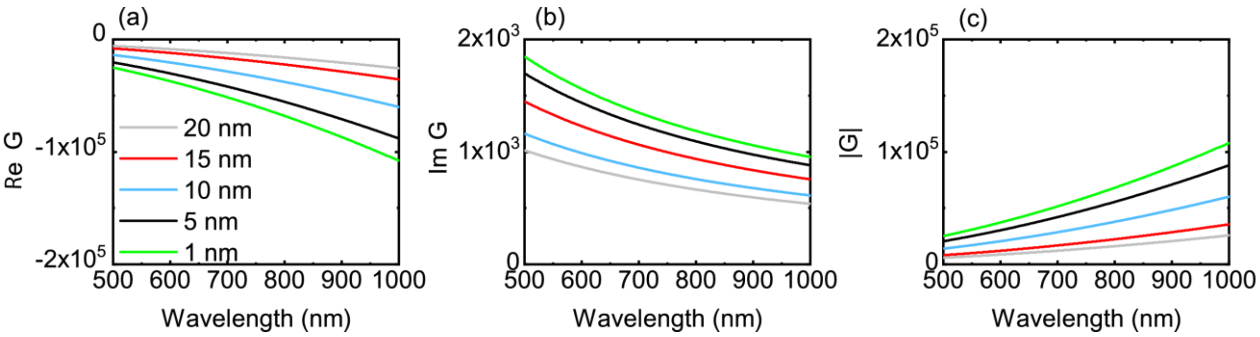

Supplementary Figure 1. (a) Real, (b) imaginary, and (c) magnitude of the dyadic Green's function describing near-field coupling in Ni/SiO_2/Au dimers.

3. FDTD simulations of reflectance and magneto-optical Kerr effect spectra

To simulate the optical and magneto-optical response of dimer lattices and Ni nanodisk arrays, we placed an electric field monitor 2 μm above the nanoparticles. Supplementary Fig. 2 shows spectra for dimers and Ni nanodisks with a diameter of 110 nm. All disks are 15 nm thick and 15 nm SiO_2 separates the Ni and Au in the dimers. The lattice constant is 400 nm. Based on these data, we selected λ = 780 nm for simulations of electric field distributions in the near field (Fig. 6 of the main manuscript).

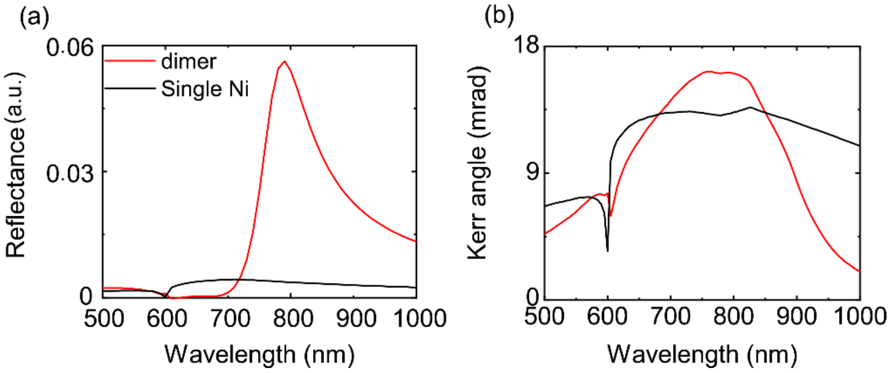

Supplementary Figure 2. FDTD simulations of (a) optical reflectance and (b) magneto-optical Kerr angle spectra for square arrays of Ni/SiO_2/Au dimers and Ni nanodisks.

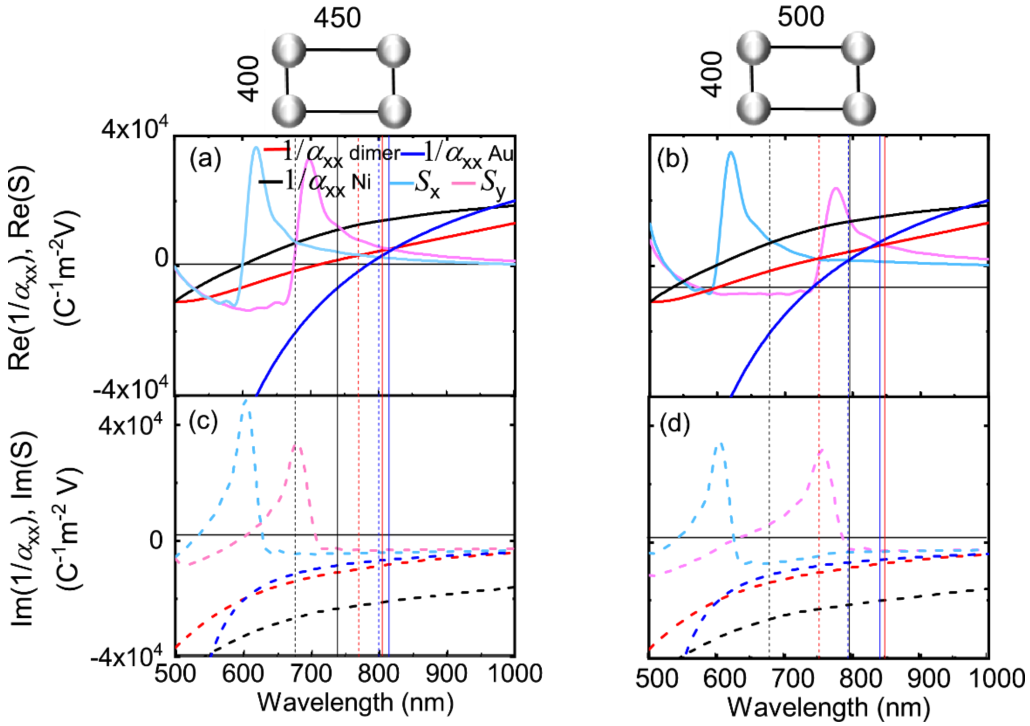

Supplementary Figure 3. (a,b) Real and (c,d) imaginary parts of $1/\alpha_{xx}$, S_x, and S_y for two rectangular lattices of Ni/SiO_2/Au dimers and Ni and Au nanodisks. The expected wavelengths of SLR modes visible in optical reflectance spectra ($\text{Re}(1/\alpha_{xx}) - \text{Re}(S_x) = 0$) and magneto-optical Kerr measurements ($\text{Re}(1/\alpha_{xx}) - \text{Re}(S_y) = 0$) of the different lattices are indicated with dashed and solid lines, respectively.

4. Optical and magneto-optical response of rectangular lattices.

Following the same analysis as for square arrays, we plot the relevant parameters $1/\alpha_{xx}$, S_x, and S_y in Supplementary Fig. 3 for lattices with $a_x = 450$ nm/$a_y = 400$ nm and $a_x = 500$ nm/$a_y = 400$ nm. Since $a_x \neq a_y$, the two lattice factors peak at different wavelengths. The real part of S_x peaks when $\lambda = na_y = 608$ nm, while S_y peaks at $\lambda = 684$ nm for $a_x = 450$ nm and $\lambda = 760$ nm for $a_x = 500$ nm.

According to Eq. 12 in the main manuscript, a strong enhancement of the optical reflectance occurs when the $\text{Re}(1/\alpha_{xx})$ and $\text{Re}(S_x)$ curves cross and $\text{Im}(1/\alpha_{xx}) - \text{Im}(S_x)$ is small. For rectangular arrays and an incident electric field along x, an SLR mode corresponding to the lattice period along y is generated. Experimental and calculated reflectance spectra on rectangular arrays of Ni/SiO_2/Au dimers, Au nanodisks, and Ni nanodisks confirm this (Supplementary Fig. 4).

The shape of magneto-optical spectra depends on the ratio between off-diagonal and diagonal effective polarizability terms. Using Eqs. 12 and 13 from the main manuscript, this gives

$$\Phi = \left| \frac{\alpha_{xy}}{\alpha_{xx}\alpha_{yy}\left(\frac{1}{\alpha_{xx}} - S_y\right)} \right|. \quad \text{(S4)}$$

SLR modes corresponding to the lattice period along *x* do therefore enhances the magneto-optical activity, as illustrated by the data in Supplementary Figs. 5(a,b) and 5(e,f).

Finally, we note that the strength of the magneto-optical dipole in periodic arrays depends on the off-diagonal component of the effective polarizability (Eq. 13 in the main manuscript). It thus depends on both lattice factors. The appearance of two DOs in the experimentally derived and calculated spectra of Supplementary Figs. 5(c,d) and 5(g,h) confirms this.

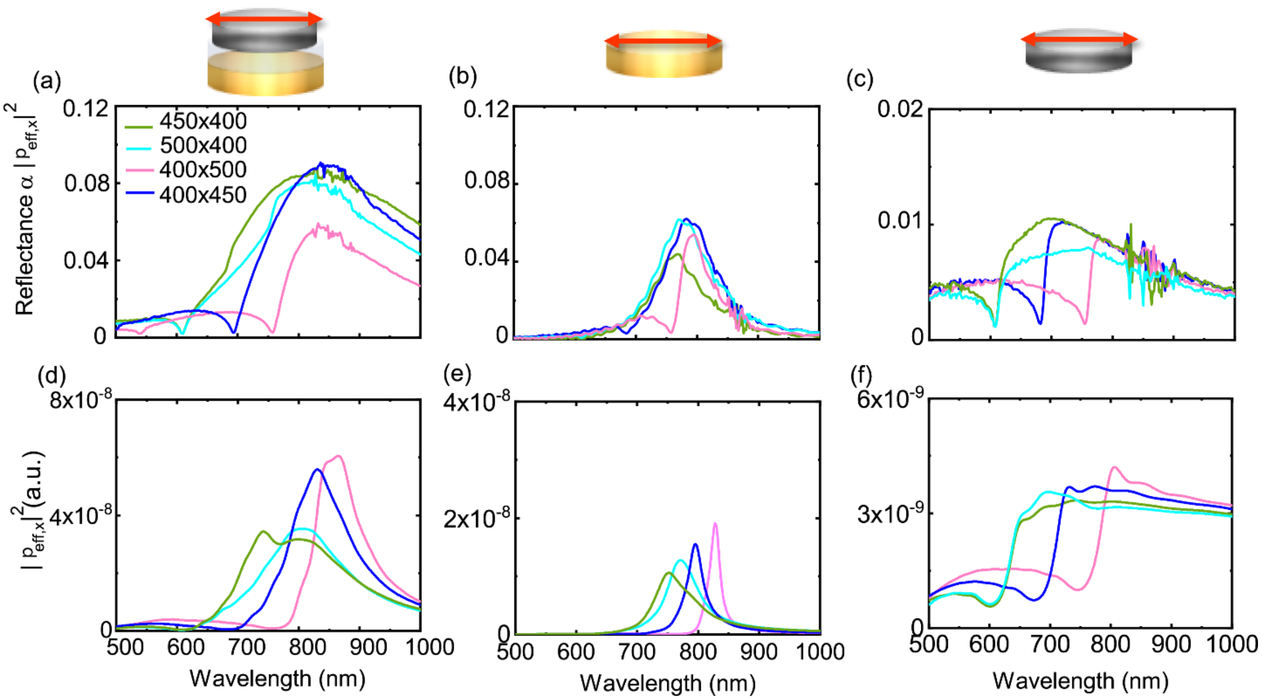

Supplementary Figure 4. Optical reflectance of rectangular arrays of (a) Ni/SiO_2/Au dimers, (b) Au nanodisks, and (c) Ni nanodisks. (d-f) Corresponding calculations of $|p_{\mathrm{eff,x}}|^2$ for the same lattices.

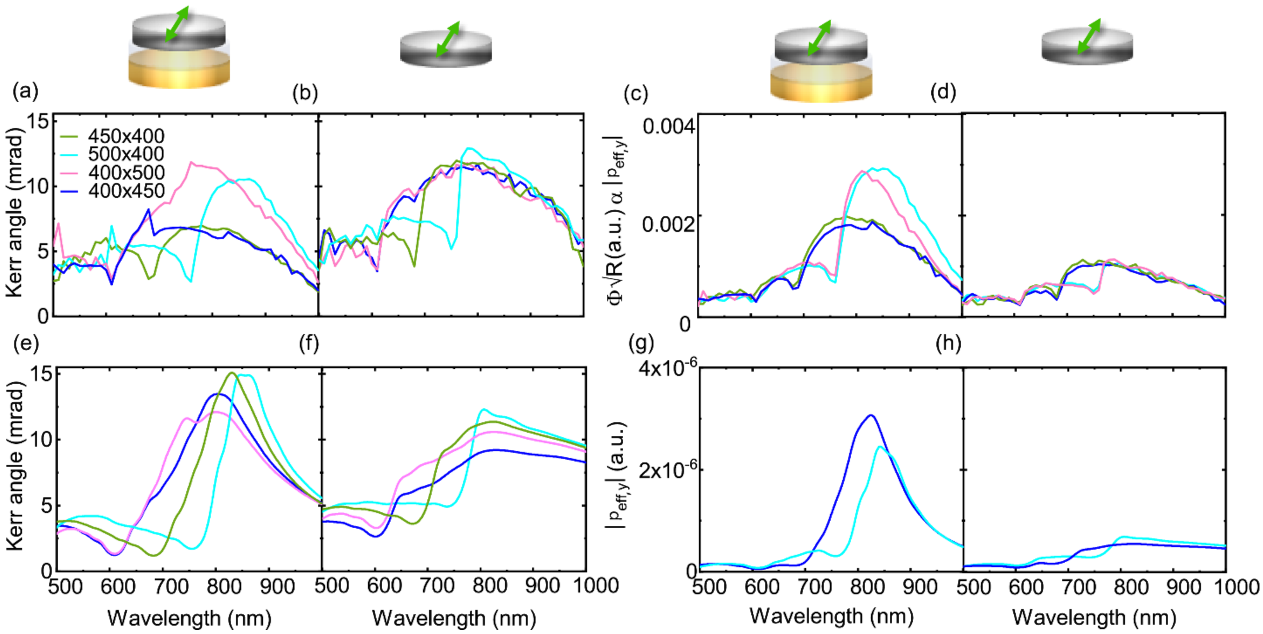

Supplementary Figure 5. (a,b) Magneto-optical Kerr angle (Φ) of rectangular arrays of (a) Ni/SiO_2/Au dimers and (b) Ni nanodisks. (c,d) Extracted values of $\Phi\sqrt{R}$ for the same lattices. This parameter, which is obtained from data in (a,b) and Supplementary Figs. 3(a,c), is proportional to the effective magneto-optical dipole ($|p_{\mathrm{eff},y}|$). (e-h) Calculations of the magneto-optical Kerr angle ($|p_{\mathrm{eff},y}|/|p_{\mathrm{eff},x}|$) and $|p_{\mathrm{eff},y}|$ for Ni/SiO_2/Au dimer and Ni nanodisk arrays.
